# Constructing Lipid-Like Biomimetic Structure via Electrolyte Designation for Stable Zinc-Ion Batteries

**DOI:** 10.1021/acsnano.4c18796

**Published:** 2025-04-07

**Authors:** Zhuoxi Wu, Shuo Yang, Zhiquan Wei, Yiqiao Wang, Xinru Yang, Jiaxiong Zhu, Hu Hong, Pei Li, Xue-Feng Yu, Chao Peng, Chunyi Zhi

**Affiliations:** † Department of Materials Science and Engineering, City University of Hong Kong, Kowloon 999077, Hong Kong SAR, China; ‡ Materials Interfaces Center, Shenzhen Institute of Advanced Technology, Chinese Academy of Sciences, Shenzhen 518055, Guangdong, China; § Hong Kong Institute for Advanced Study, City University of Hong Kong, Kowloon 999077, Hong Kong SAR, China; ∥ Hong Kong Institute for Clean Energy, City University of Hong Kong, Kowloon 999077, Hong Kong SAR, China; ⊥ Centre for Advanced Nuclear Safety and Sustainable Development, City University of Hong Kong, Kowloon 999077, Hong Kong SAR, China

**Keywords:** zinc-ion batteries, biomimetic electrolyte, zinc anode, high Coulombic efficiency, artificial protective layer

## Abstract

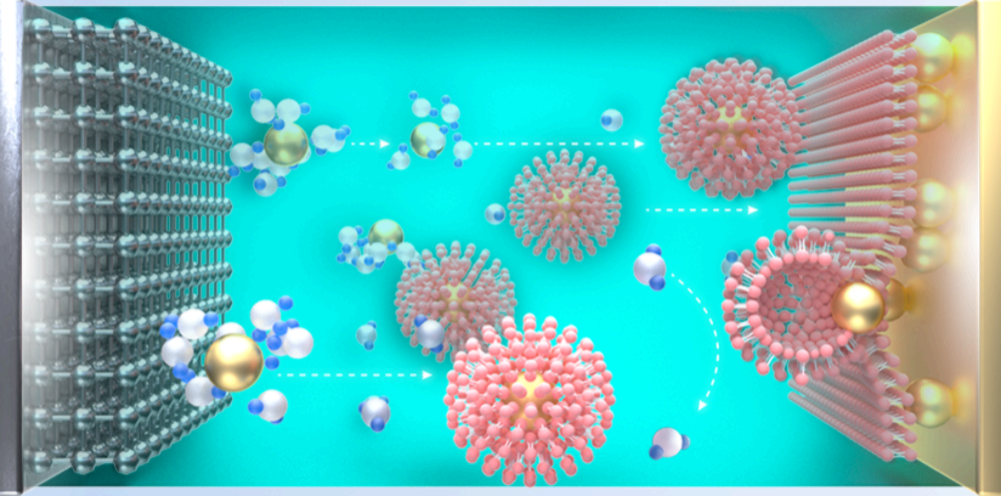

Zinc-ion batteries (ZIBs) have attracted widespread attention in recent years. However, due to the aqueous electrolyte’s high activity, the zinc anode is affected by severe side reactions such as corrosion and hydrogen evolution, resulting in poor reversibility. Inspired by the structure of a lipid bilayer in biology, in this paper, we introduce lithium nonafluorobutylsulfonate to inhibit the water activity via vigorous binding between S=O and H_2_O and form a bilayer lipid-like protective structure on the surface of the zinc anode, thereby improving the reversibility of the zinc anode and extending the lifespan of the ZIBs. The zinc anode in the biomimetic electrolyte demonstrated outstanding reversibility with a 880 h cycle life and 99.91% average Comlombic efficiency in the Zn||Cu asymmetric battery, as well as a 2460 h cycle life and a cumulative capacity of 6 Ah cm^–2^ in the Zn||Zn symmetric battery (5 mA cm^–2^ and 5 mAh cm^–2^). In addition, full cells with Zn_0.25_V_2_O_5_·*n*H_2_O and MnO_2_ show excellent capacity retention of 91.67% after 1200 cycles and 100% after 1000 cycles, respectively. After cycles, the ampere-hour-level pouch cell showed a capacity retention rate of 93%. This method provides a biomimetic strategy for constructing biomimetic electrolytes to improve the reversibility of zinc anodes.

## 1 Introduction

Zinc-ion batteries (ZIBs) have received widespread attention due to their advantages of high volumetric energy density, low cost, and high safety.^1−5^ However, in recent years, the development of ZIBs has encountered bottlenecks, mainly due to the poor reversibility of the zinc metal anode resulting from dendritic deposition. Dendritic deposition in ZIBs comes from enormous side reactions such as corrosion and hydrogen evolution caused by H_2_O decomposition on the anode surface.^6,7^ In conventional electrolytes, Zn^2+^ combines with H_2_O to form a solvent structure of Zn(H_2_O)_6_^2+^. During the electrochemical process, because the zinc anode is exposed to the electrolyte without protection along with the Zn^2+^ plating/stripping, active H_2_O in the solvation sheath decomposes on the surface of the zinc anode.^8,9^ This process produces a large number of irreversible byproducts that accumulate unevenly on the electrode surface, forming a series of raised byproduct hills.^10−12^ In the process, Zn^2+^ is consumed to generate irreversible byproducts, leading to low Coulombic efficiency (CE).^13^ More importantly, driven by the “tip effect”, Zn^2+^ preferentially nucleates and grows on these protrusions, eventually turning into dendrites that pierce the separator and cause the battery to short-circuit.^14,15^ Therefore, it remains a challenge to effectively improve the reversibility of the zinc anode and inhibit the decomposition of water on the surface of the zinc anode.^16,17^

In the past decade, many valuable strategies have been proposed to protect zinc anodes. Among them, the two most widely used methods are to limit free H_2_O molecules in the electrolyte via hydrogen bonding between additives and water and surface engineering on the zinc anode [including the artificial solid electrolyte interphase (SEI) and spontaneously formed SEI].^18,19^ For example, Wang et al. introduced xylitol into the ZnSO_4_ electrolyte to regulate the electrolyte structure by forming hydrogen bonds between xylitol molecules and H_2_O, thus limiting the water activity.^20^ Besides, Wang and co-workers constructed an artificial SEI of a gradient ZnF_2_–Zn_3_(PO_4_)_2_ interphase on the surface of the zinc anode, thereby boosting the reversibility of the zinc anode.^21^ Both methods can effectively protect the zinc anode from parasitic reactions during the electrochemical process. Although utilizing a single strategy (limiting free water or zinc anode surface engineering) has achieved decent results, combining the two approaches may bring more significant performance improvements.^22^ Constructing a kind of electrolyte that can simultaneously suppress active H_2_O and protect the zinc anode seems to be an effective way of boosting the reversibility of zinc in ZIBs.

A lipid bilayer is a typical structure in biology.^23^ In cells, it serves as the main component of the cell membrane, protecting the stability of the internal structure of the cell; in intercellular material transport, it participates in the formation of vesicles to prevent the transported materials from being invaded by cell fluid impurities.^24^ The lipid bilayers that make up cell membranes and vesicles contain hydrophilic heads and hydrophobic tails, so this structure seems likely constructible in an electrolyte.

Inspired by the lipid bilayer, here, taking advantage of the hydrophilicity of the sulfonic acid group and the hydrophobicity of the fluorine-containing group, we introduced lithium nonafluorobutanesulfonate (LiFBS) to 3 M Zn(OTF)_2_ electrolyte to modify the electrolyte. The hydrophilic −SO_3_^–^ group forms a strong O–H bond with the H_2_O molecules in the electrolyte, limiting the activity of free H_2_O molecules.^25^ More importantly, the FBS anions also tightly absorb the zinc metal anode, forming a bilayer lipid-like protective structure. The inner hydrophobic fluorine-containing group of the bilayer resists the passage of H_2_O molecules, thereby protecting the zinc metal anode from active H_2_O. Thanks to these effects, the reversibility of a zinc anode with a biomimetic electrolyte in both Zn||Cu asymmetric cells and Zn||Zn symmetric cells is significantly improved. Full batteries with biometic electrolytes also show high capacity retention and excellent storage performance.

## 2 Result and Discussion

### Electrolyte Characterization

**Figure 1:**
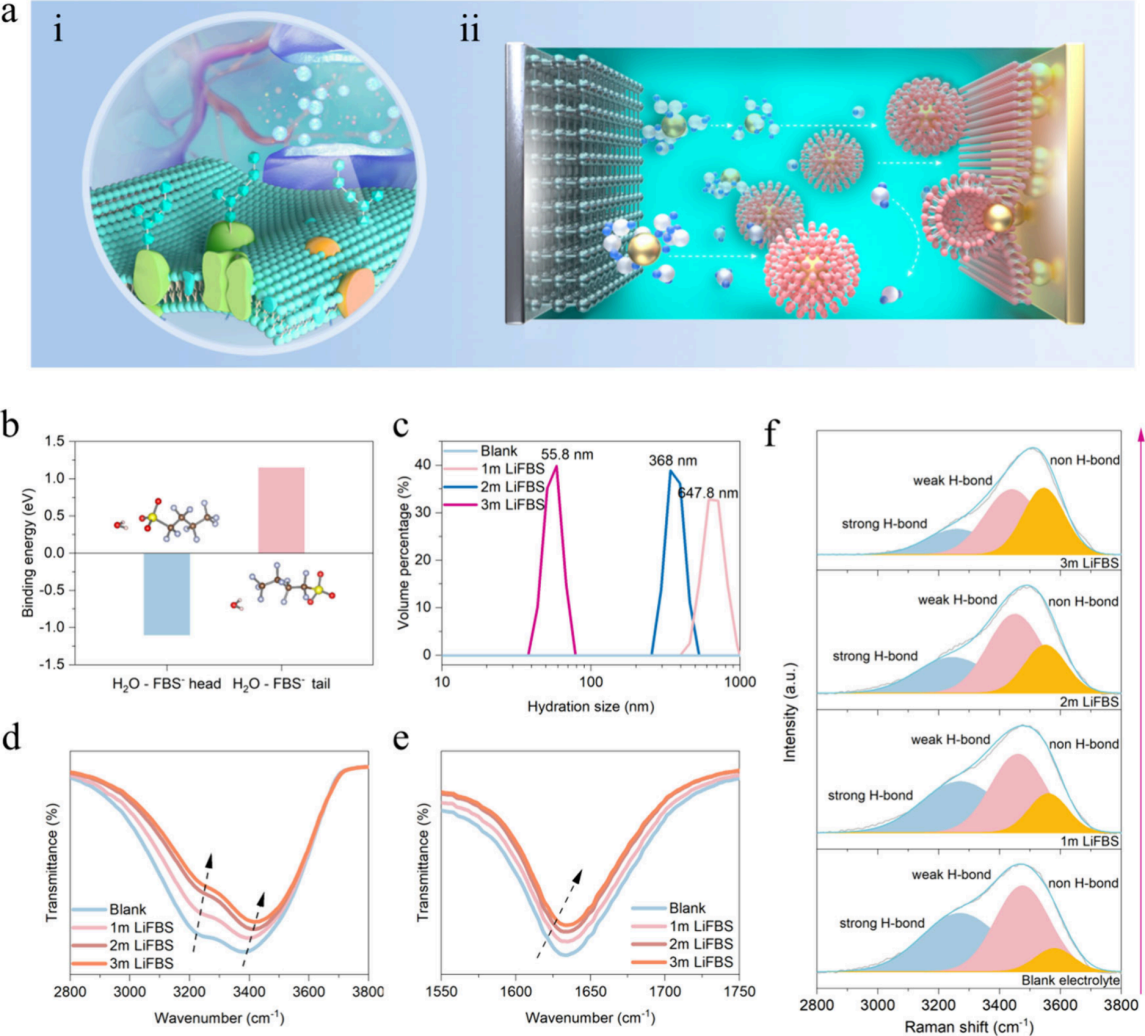
Schematic diagram of the lipid bilayer and the mechanism of a biomimetic electrolyte and characterization of the biomimetic electrolyte. (a) (i) Lipid bilayer structure in biology and (ii) mechanism of a biomimetic electrolyte in the battery. (b) Binding energy between the H_2_O–FBS head and H_2_O–FBS tail. (c) DLS analysis for the size of FBS ionic clusters in 3 M Zn(OTF)_2_ and 3 M Zn(OTF)_2_ + *x* M LiFBS (*x* = 1–3). (d and e) FTIR analysis of the water activity limited by ionic clusters formed by FBS and H_2_O. (f) Raman spectra of hydrogen signals in different concentrations of LiFBS.

Synapses are essential signal transmission structures in organisms. The cell membrane, composed of a lipid bilayer, protects cells from external influences. At the same time, another structure of vesicles, also composed of a lipid bilayer, allows substances to be quickly transmitted between cells without interference [Figure 1a(i)]. In the experiment, LiFBS was added to a 3 M Zn(OTF)_2_ electrolyte to build similar structures in batteries. Thanks to the hydrophilicity of the sulfonic acid (−SO_3_) group and the natural hydrophobicity of the fluorine group (−C_4_F_9_), the FBS anions will be distributed in the electrolyte in a tail-to-tail manner. Among the electrolytes, the hydrophobic fluorine-containing groups closely adhere to each other through strong F–F interaction; meanwhile, the hydrophilic sulfonic acid (−SO^3–^) groups are located on the inner and outer surfaces, forming vesicle-like clusters [Figure 1a(ii)]. The outer −SO^3–^ groups will strongly bind with free H_2_O in the electrolyte. As a result, free H_2_O is anchored to the outer layer surface. More importantly, the FBS anions also exhibit strong adsorption with zinc metal, thanks to which a lipid-bilayer-like protective structure is formed on the zinc anode surface [Figure 1a(ii)]. The free H_2_O molecules approaching the electrode surface along with Zn^2+^ will be tightly restricted by the outer −SO_3_ groups and cannot pass through the protective layer due to the strong repulsion of the fluorinated groups. At the same time, Zn^2+^ can shuttle freely, which ensures the fast transport kinetics of Zn^2+^ on the anode surface and suppresses the adverse effects of free H_2_O.

First, to confirm our conjecture that FBS interacts strongly with water, we calculated the binding energy between FBS and H_2_O. As shown in Figure 1b, the binding energy between the head of FBS and H_2_O reaches −1.24 eV, while the binding energy between the tail of FBS and H_2_O is 1.28 eV. Dynamic light scattering (DLS) was employed to analyze the ionic group behavior of electrolytes with different concentrations of LiFBS (Figure 1c). The results showed that the size of micelle-like ionic clusters turned smaller from 647.8 to 55.8 nm as the concentration of LiFBS increased. This is because FBS exhibits surfactant-like properties (the FBS anion has a hydrophilic head and a hydrophobic tail). As the concentration increases, the large clusters formed break down into smaller clusters, similar to the formation of micelle solutions. Fourier transform infrared (FTIR) and Raman spectroscopy are commonly used to characterize the strength of chemical bonds.^26^ Here, we studied the activity of H_2_O in the electrolyte through FTIR and Raman spectroscopy. As shown in Figure 1d,e, as the LiFBS content increases, the O–H bond absorption intensity in the FTIR spectra (around 3400, 3200, and 1600 cm^–1^) decreases, exhibiting an overall blue shift trend. This is due to the strong interaction between the FBS anions and H_2_O, which weakens the O–H vibration and the hydrogen bond between the free H_2_O.

Besides, in Raman spectroscopy, the peak of the O–H bond can be contributed by a strong H-bond, weak H-bond, and non-H-bond. When the H-bond in water weakens, the vibration of O–H increases, resulting in a blue shift in the Raman spectra. It can be seen from Figure 1f that a strong H-bond is dominant in the blank electrolyte. At the same time, a weak H-bond occupies a small part, and there are almost no H_2_O molecules in the state of non-H-bond, which confirms the existence of a large number of strong H-bonds in the blank aqueous electrolytes mentioned above. As the LiFBS content increases, the strong H-bonds (3250 cm^–1^) between H_2_O molecules gradually transform into a weak H-bond (3400 cm^–1^) and a non-H-bond (3550 cm^–1^), indicating the firm anchoring of the FBS group and H_2_O and confirming the breakage of pristine H-bond networks. Finally, in the biomimetic electrolyte (3 M LiFBS), most of the strong H-bond is transformed into a weak H-bond and a non-H-bond, and the latter two dominate, revealing that the conventional H-bond network formed between H_2_O molecules is broken. Echoed by the binding energy results, FTIR and Raman spectra demonstrate the strong affinity of the −SO_3_^–^ groups in FBS for water, significantly reducing the electrolyte’s water activity. These results also confirm our view that FBS-induced ionic clusters can substantially inhibit the activity of free H_2_O in the electrolyte. Furthermore, we employed NMR spectroscopy to characterize the biomimetic electrolyte. The results showed that, as the concentration of LiFBS increased from 1 to 3 M, the chemical shift of ^1^H in H_2_O moved from 4.703 ppm in the blank electrolyte to 4.687, 4.681, and 4.676 ppm (Figure S1). This can be ascribed to the fact that, as the concentration of FBS increased, the binding between the sulfonic acid group and the H_2_O molecule became more vigorous, resulting in a lower degree of freedom of water.

Besides, as the FBS anion concentration increases, the Zn^2+^ solvation structure gradually transforms from Zn^2+^ (H_2_O)_6_ to Zn^2+^(OTF^–^)_1.4_(FBS)_0.4_(H_2_O)_4.2_, Zn^2+^(OTF^–^)_1.3_(FBS)_0.7_(H_2_O)_4_, and Zn^2+^(OTF^–^)_1.2_(FBS)_1.0_(H_2_O)_3.8_ (for 1 M LiFBS, 2 M LiFBS, and 3 M LiFBS, respectively; Figure S3). As the FBS anion concentration increases, the water content in the Zn^2+^ solvation sheath gradually decreases but is not entirely replaced. The reason may be that the added LiFBS preferentially spontaneously forms a lipid-bilayer-like structure on the zinc anode, with only a small part of the FBS anions attacking the Zn^2+^ solvation sheath. Besides, the density functional theory (DFT) binding energy calculations indicate that the interaction between Zn and FBS is stronger than that between Zn and H_2_O (Figure S4). This suggests that the lower concentration of FBS in the zinc-ion solvation sheath is due to the preferential participation of FBS anions in forming the protective layer on the zinc anode surface rather than competing for coordination.

**Figure 2:**
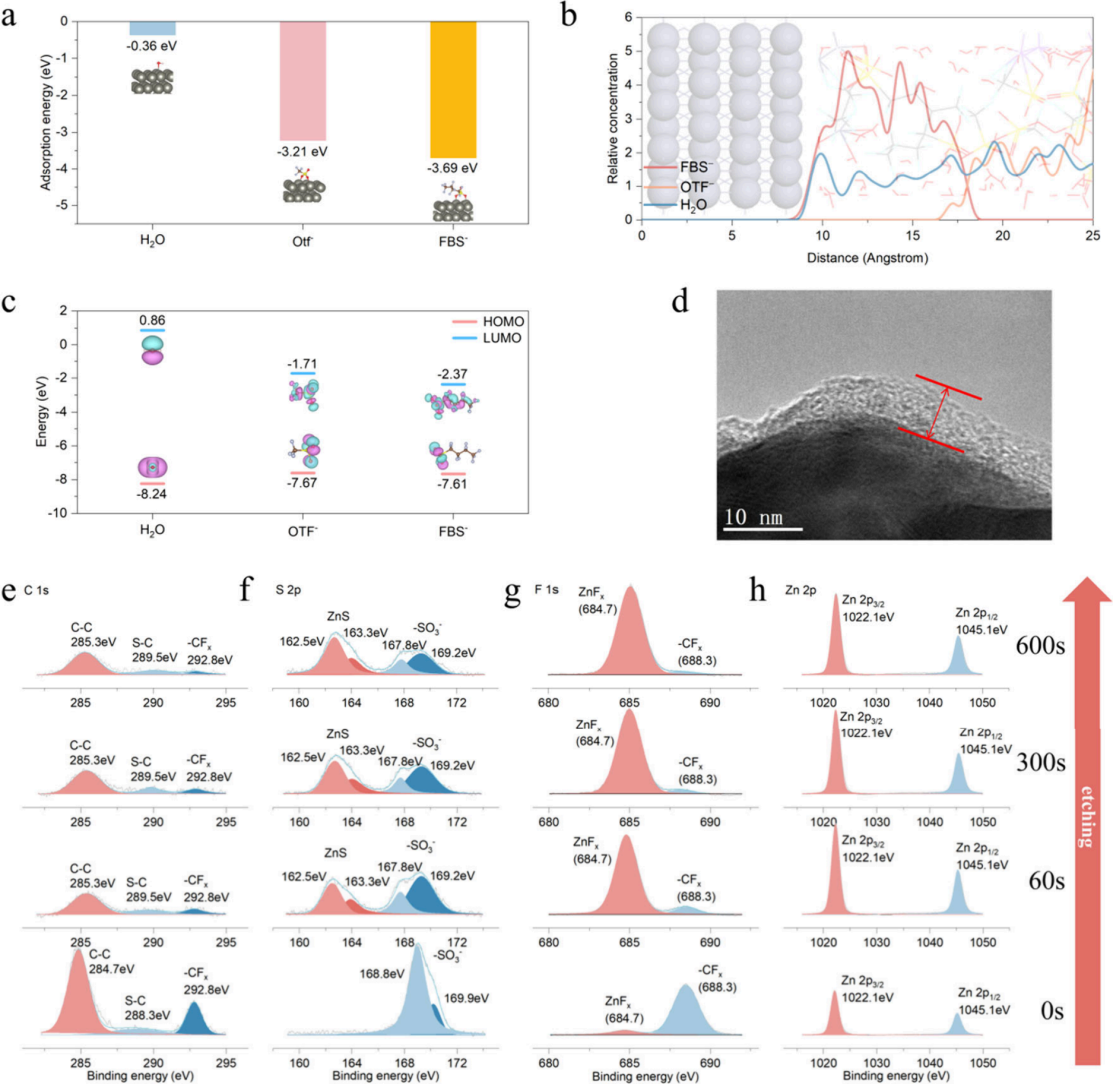
Schematic of SEI formation on the zinc anode. (a) Adsorption energies of H_2_O, OTF^–^, and FBS on a zinc metal anode. (b) Relative concentrations of H_2_O, OTF^–^, and FBS on the surface of a zinc anode. (c) HOMO/LUMO energies of H_2_O, OTF^–^, and FBS. (d) TEM image of the formed SEI on the zinc anode after cycles. (e and f) XPS spectra of C, S, F, and Zn of the SEI.

The FBS anions also exhibit strong adsorption with the zinc anode. DFT is carried out to calculate the adsorption energy of different anions or molecules to the zinc anode. Results show that the adsorption energy of FBS anions to zinc anode (−3.69 eV) is much larger than those of both OTF^–^ (−3.21 eV) and H_2_O (−0.36 eV) (Figure 2a). This characteristic causes the FBS anions to form a bilayer protective structure on the zinc anode surface (because the fluorinate containing group is hydrophobic and would not contact water in the electrolyte; Figure 2a). This bilayer protective structure is laid flat on the zinc anode surface. To verify this opinion further, we evaluated the relative concentration distribution of different ions on the zinc anode surface. The relative concentration distribution curve shows that FBS anions account for the majority of the distribution on the surface of the zinc anode, and the two peaks on the relative concentration curve also echo the proposed bilayer structure (Figure 2b). This result further confirms our hypothesis. Due to the strong zinc philicity and hydrophobicity, during the zinc deposition process, Zn^2+^ freely passed through the bilayer under the action of electric field force and concentration difference, achieving uniform zinc deposition, while free H_2_O was trapped on the −SO_3_^–^ groups of the outer bilayer surface due to its strong anchoring effect with −SO_3_^–^ groups and the repulsive effect of the −CF_*x*_ group in the inner layer [Figure 1a(ii)]. Notably, the adsorption of FBS and OTF on the zinc anode surface is significantly stronger than that of H_2_O. However, due to the competitive interaction between FBS and OTF on the zinc anode surface, as well as the large steric hindrance of FBS anions, OTF is repelled to the outer layer. In contrast, H_2_O molecules are not entirely excluded due to their smaller size. Nevertheless, the adsorbed particles near the zinc anode surface are primarily FBS. The formed lipid-bilayer-like structure successfully isolates the direct contact between water and the zinc anode.

In addition, the HOMO/LUMO energies of FBS, OTF^–^, and H_2_O are also calculated to infer whether SEI will be generated. As shown in Figure 2c, the results show that FBS has higher HOMO energy, which means, during the zinc deposition process, the bilayer structure may decompose to form a protective SEI. In order to investigate whether SEI is formed, we deposited zinc on a transmission electron microscopy (TEM) copper grid and employed TEM to observe.^27^ As we expected, an evident amorphous band on the surface of the zinc crystal was observed, which can be considered to be the SEI structure (Figure 2d). Notably, a stable SEI was not observed in zinc that cycled in the blank electrolyte (Figure S6). This result indicates that the stable SEI in biomimetic electrolytes may come from the partial degradation of the lipid-bilayer-like protective layer during the electrochemical process. XPS was carried out to analyze the SEI components further.^28,29^ In the C 1s spectrum, the surface consists of C–C (284.7 eV), S–C (288.3 eV), and −CF_*x*_ (292.8 eV), which may originate from the FBS anions. As the etching time increases, the peaks of S–C and −CF_*x*_ weaken significantly and almost disappear, and the peak of C–C also gradually weakens, proving that the outer SEI is composed of polyanions degraded from FBS (Figure 2e). In the S 2p spectrum, the surface composition is −SO_3_^–^ groups (168.8 and 169.9 eV). As the etching time increases, the signal of −SO_3_^–^ weakens significantly. In contrast, the signal of ZnS_*x*_ gradually increases (162.5 and 163.3 eV; Figure 2f), indicating that the S-containing groups are decomposed near the zinc anode and combine with Zn^2+^ to form ZnS_*x*_. The F 1s spectrum also shows a similar trend in which, at the surface, F is mainly bonded with carbon (−CF_*x*_, 688.3 eV). As the etching depth increases, the −CF_*x*_ signal gradually disappears, transforming to a strong ZnF_*x*_ signal (684.7 eV), which indicates that organic fluoride existed in the outer SEI and inorganic ZnF_*x*_ existed in the inner SEI (Figure 2g). The Zn 2p spectrum shows that there are fewer Zn-containing inorganic components on the SEI surface (Figure 2h). As the etching time increases, the spectrum signal increases, proving the increase in Zn-containing inorganic compounds and further confirming the conclusion that ZnS_*x*_ and ZnF_*x*_ form in the inner SEI. The result of atomic proportion depending on the etching time also shows that C atoms are mainly distributed within the range of the former 60 s etching time, while after an etching time of 60 s, S and F atoms account for the majority of the proportion (Figure S5). XPS analysis demonstrates that the SEI mainly comprises organic polyanions in the outer layer and inorganic components (ZnS_*x*_ and ZnF_*x*_) in the inner layer. The polyanions–ZnS_*x*_–ZnF_*x*_ SEI protect the zinc anode from being exposed to the electrolyte and side reactions caused by H_2_O decomposition further, avoiding the consumption of zinc by irreversible side reactions, thereby improving the reversibility of the zinc anode. Moreover, the inorganic ZnS_*x*_ and ZnF_*x*_ components ensure rapid Zn^2+^ diffusion during the electrochemical process while also repulsing free shuttling of H_2_O.

### Side Reaction Inhibition

**Figure 3:**
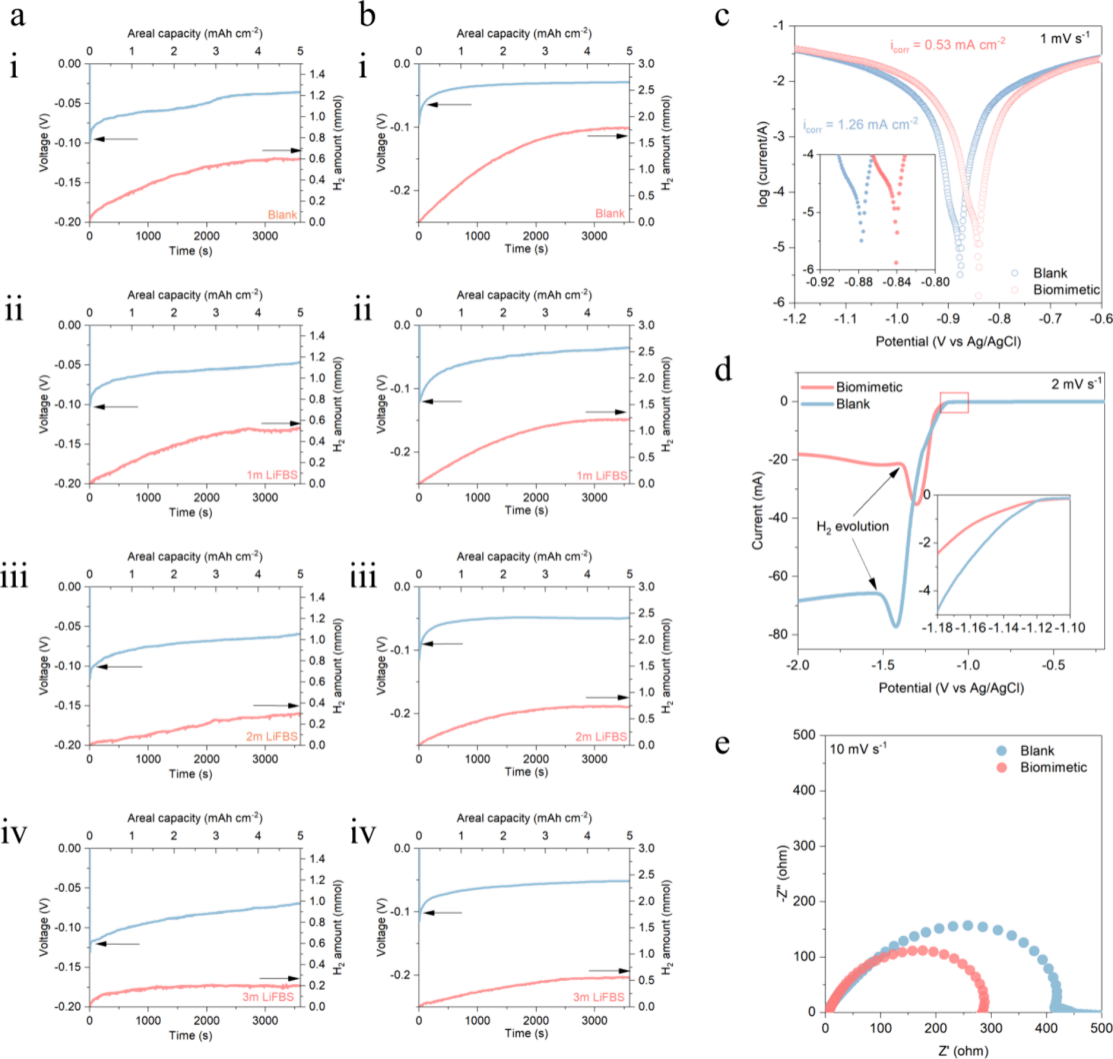
Hydrogen evolution from side reactions on the zinc anode and electrolytes (together with the discharge voltage profile). [a(i–iv)] Hydrogen gas evolution from the first cycle of zinc deposition on a Cu substrate in a blank electrolyte and 3 M Zn(OTF)_2_ + *x* M LiFBS (*x* = 1–3) electrolyte at 25 °C (upper curve, discharge voltage profile of a Zn||Cu half-cell at 5 mA cm^–2^ and 5 mAh cm^–1^; lower curve, hydrogen evolution curve). [b(i–iv)] Hydrogen gas evolution from the first cycle of zinc deposition on a Cu substrate in a blank electrolyte and 3 M Zn(OTF)_2_ + *x* M LiFBS (*x* = 1–3) electrolyte at 45 °C. (c) Tafel curves of a blank electrolyte and a biomimetic electrolyte (the inset is the partial enlargement between −0.92 and −0.80 V). (d) LSV comparison of a blank electrolyte and a biomimetic electrolyte (the inset is the partial enlargement between −1.18 and −1.10 V). (e) EIS of Zn||Zn symmetric cells with a blank electrolyte and a biomimetic electrolyte.

To concretely characterize the inhibition of water activity by FBS ionic clusters and the protective effect of the bilayer structure on the zinc anode, we set up a gas-pressure-sensing device to test the gas production of the zinc anode (the device structure is provided in Figure S7).^22^Figure 3a(i–iv) shows the gas production of the first cycle of discharge of Zn||Cu asymmetric cells at 25 °C with different FBS anion concentrations. It can be seen that, as the FBS anion concentration increased, the gas production of the Zn||Cu asymmetric cells dropped from the initial 0.605 mmol to 0.52, 0.315, and 0.195 mmol, with decreasing percentages of 14.2%, 48%, and 67.8%, respectively. In order to further verify the suppressive effect of biomimetic electrolytes on side reactions, we conducted gas production tests under more stringent conditions at 45 °C with the Zn||Cu asymmetric cells. The results are shown in Figure 3b(i–iv). The amount of hydrogen produced also gradually decreases with increased FBS anion concentration. Compared with the blank electrolyte (1.785 mmol), the gas evolution amount of the Zn||Cu asymmetric cell using a reverse micellar electrolyte (3 mol of FBS) is only 0.565 mmol, indicating a decrease of 68.4%. These results prove that the biomimetic electrolyte plays a vital role in inhibiting hydrogen evolution and causing a series of parasite reactions, which can be undoubtedly attributed to the aforementioned anchoring effect of FBS-induced ionic clusters on water and the isolation of H_2_O molecules by the protective layer on the surface of the zinc anode.

After measuring the gas production on the electrode surface, we further characterized corrosion inhibition of the anode by the lipid-bilayer structure. The Tafel curve shows that the equilibrium potential of the zinc anode in the electrolyte shifted positively from −0.88 to −0.84 V, and the calculated corrosion current density of the electrode decreased from 1.26 to 0.53 mA cm^–2^, which means that the spontaneous corrosion of the electrode was greatly suppressed (Figure 3c). In addition, it was also observed on the linear-sweep voltammetry (LSV) curve that the H_2_ evolution potential becomes more negative. The corrosion current is significantly reduced, confirming the function of the bilayer protective structure on the zinc surface in HER inhibition (Figure 3d). These results demonstrate that the spontaneous irreversible side reactions at the zinc anode are greatly suppressed, verifying the effectiveness of the biomimetic electrolyte. In order to confirm that the lipid-bilayer protection structure can bring faster zinc-ion transmission to the zinc anode surface, we performed electrochemical impedance spectroscopy (EIS) tests. The results showed that the battery impedance in the biomimetic electrolyte was greatly reduced (Figure 3e). These results verified our previous conjecture that the phospholipid bilayer protection structure on the anode surface would prevent free H_2_O from approaching the zinc anode surface, while allowing zinc ions to pass quickly. In addition, we also studied the nucleation and growth process of Zn^2+^ on the surface of the zinc anode via the chronoamperometry method. The results showed that, compared with the blank electrolyte, the current increase in the biomimetic electrolyte was greatly slowed in the first 50 s and the subsequent current also decreased, which indicates that the disordered 2D diffusion of Zn^2+^ in the initial nucleation stage was greatly suppressed and the 3D diffusion of Zn^2+^ dominated (Figure S8). The result shows that the protective layer can inhibit the side reactions caused by water decomposition and regulate the zinc flux to create orderly zinc deposition.

### Symmetric Cell and Half-Cell Performance

**Figure 4:**
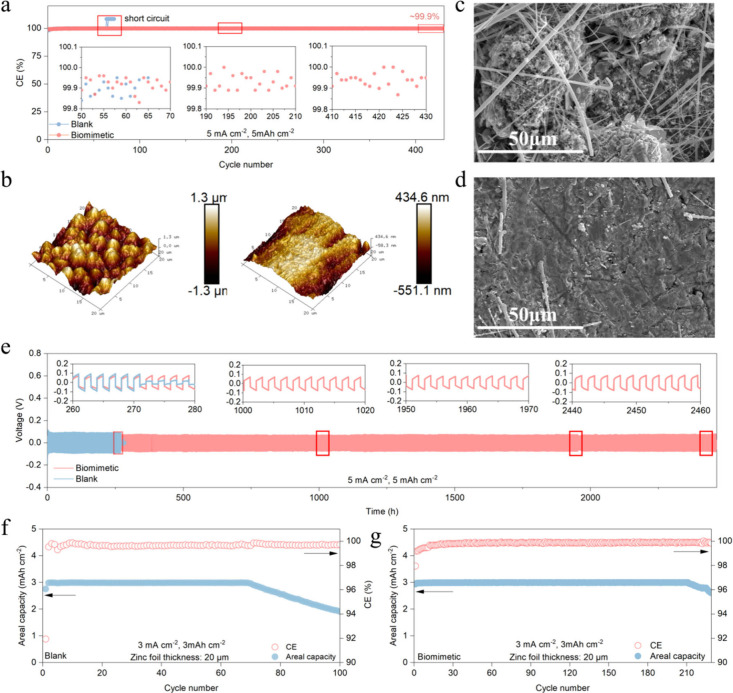
Improvement of the zinc anode in a biomimetic electrolyte. (a) CE comparison of the blank and biomimetic electrolytes of Zn||Cu asymmetric cells at 5 mA cm^–2^ and 5 mAh cm^–2^ (the insets are partial enlargements at different times). (b) AFM image of the zinc anode after cycling in a blank electrolyte (left) and a biomimetic electrolyte (right). SEM image comparison of zinc deposited on a Cu substrate with (c) blank electrolyte and (d) biomimetic electrolyte. (e) Time–voltage curves of Zn||Zn symmetric cells with blank and biomimetic electrolytes at 5 mA cm^–2^ and 5 mAh cm^–2^ (the insets show the representative voltage profiles). Inflection points on the capacity curve of Zn||Cu half-cell with (f) blank electrolyte and (g) biomimetic electrolyte.

The reversibility of the zinc anode in biomimetic electrolytes was studied via a Zn||Cu asymmetric cell. Compared with a blank electrolyte, the cycle life and CE of the Zn||Cu asymmetric cell in a biomimetic electrolyte was greatly improved, reaching a cycle life of more than 880 h at 5 mA cm^–2^ and 5 mAh cm^–2^ and a high average CE of 99.91% (Figures 4a and S9). Voltage profiles can be seen in Figure S10. In contrast, the Zn||Cu asymmetric cell with a blank electrolyte could only cycle for 127 h under the same conditions. In blank electrolytes, at a high current density, irreversible reactions guided by electric field strength will occur in large quantities on the anode surface. At the same time, at high areal capacity, due to the large amount of zinc that participated in a single cycle, more zinc participates in side reactions in the blank electrolyte. Under the synergetic effect of the two adverse effects, irreversible byproducts quickly accumulate on the surface of the zinc anode, causing the battery to short-circuit rapidly in a short time. Compared with the blank electrolyte, the anchoring effect of the H_2_O molecule in the biomimetic electrolyte and the protective bilayer significantly reduce the probability of parasite reactions on the surface of the zinc anode, allowing it to operate stably at high current density and areal capacity. We used atomic force microscopy (AFM) to observe the surface of the zinc anode after the cycle. The AFM results showed that surface deposition of the zinc anode after cycling in the blank electrolyte was very messy, with many uneven dendrite-like undulations (Figure 4b). In contrast, the zinc anode deposited in the bionic electrolyte showed a more uniform deposition surface after cycling (Figure 4b and the corresponding contour image of AFM can be seen in Figures S11 and S12). Scanning electron microscopy (SEM) was employed to observe the electrode of cycled Zn||Cu asymmetric cells. Parts c and d of Figure 4 are SEM images of zinc deposition on a Cu substrate of a blank electrolyte and a biomimetic electrolyte, respectively. From Figure 4c, we can see that, after cycling in s blank electrolyte, loose and uneven deposition is noted and a large number of byproducts are observed on the surface. As the reaction proceeds and the operation time passes, these byproducts eventually stack to form zinc dendrites, causing battery deterioration. However, after cycling in a biomimetic electrolyte, a denser deposition morphology is shown, and no byproducts are observed on the surface (Figure 4d). Moreover, X-ray diffraction (XRD) characterization shows that the zinc hydroxysulfate (Zn_4_SO_4_(OH)_6_·*x*H_2_O) byproduct was generated on the surface of the zinc anode after cycling in the blank electrolyte. On the contrary, no corresponding diffraction peak was observed in the XRD results of the zinc anode cycled in the biomimetic electrolyte (Figure S13). These results further confirm that the lipid-bilayer protection structure and the SEI it brings have a significant impact on the protection of the zinc anode. A Zn||Zn symmetrical battery was employed to study the zinc anode’s reversibility further. Similarly, the Zn||Zn symmetrical battery demonstrated the same improvement trend, with a cycle life of over 2460 h at 5 mA cm^–2^ and 5 mAh cm^–2^ (Figure 4e) and a cumulative capacity of 6 Ah cm^–2^. The lifespan of Zn||Zn symmetric cells with biomimetic electrolytes was significantly increased compared to that of blank electrolytes (271 h).

Besides, our previous work has reported on the problem of the zinc consumption rate and zinc reversibility.^30^ The consumption rate of zinc does not seem to depend entirely on CE, but it can be roughly calculated from the inflection point on the capacity curve (see the Supporting Information for the zinc consumption rate calculation). We used 20 μm zinc foil to test the Zn||Cu half-cell at 3 mA cm^–2^ and 3 mAh cm^–2^. The results are shown in Figure 4f,g. The inflection points on the capacity curves of the blank and biomimetic electrolytes were at the 71st and 212th cycles, respectively, which means that the zinc foil is depleted and insufficient to provide an areal capacity of 3 mAh cm^–2^ after such a number of cycles. The corresponding time–voltage curves can be seen in Figures S14 and S15. Figure S16 shows the voltage profiles of the first cycle, the middle cycle, and the cycle corresponding to the inflection points of the blank (Figure S16a) and biomimetic (Figure S16b) electrolytes. It is observed that the voltage polarization at the end of discharge drops sharply when the inflection point appears, which echoes zinc depletion. We calculated the average zinc consumption rate per cycle via eq 1 in the Supporting Information. The results show that the average zinc consumption rate per cycle in the blank electrolyte is 4.24%. In comparison, the zinc consumption rate per cycle in the biomimetic electrolyte is only 1.39%, which is only one-third of that of the blank electrolyte (Figure S16c). This result indicates that the lipid-bilayer-like protection structure on the surface of the zinc anode in the biomimetic electrolyte significantly improves zinc reversibility.

### Full Cell Performance

**Figure 5:**
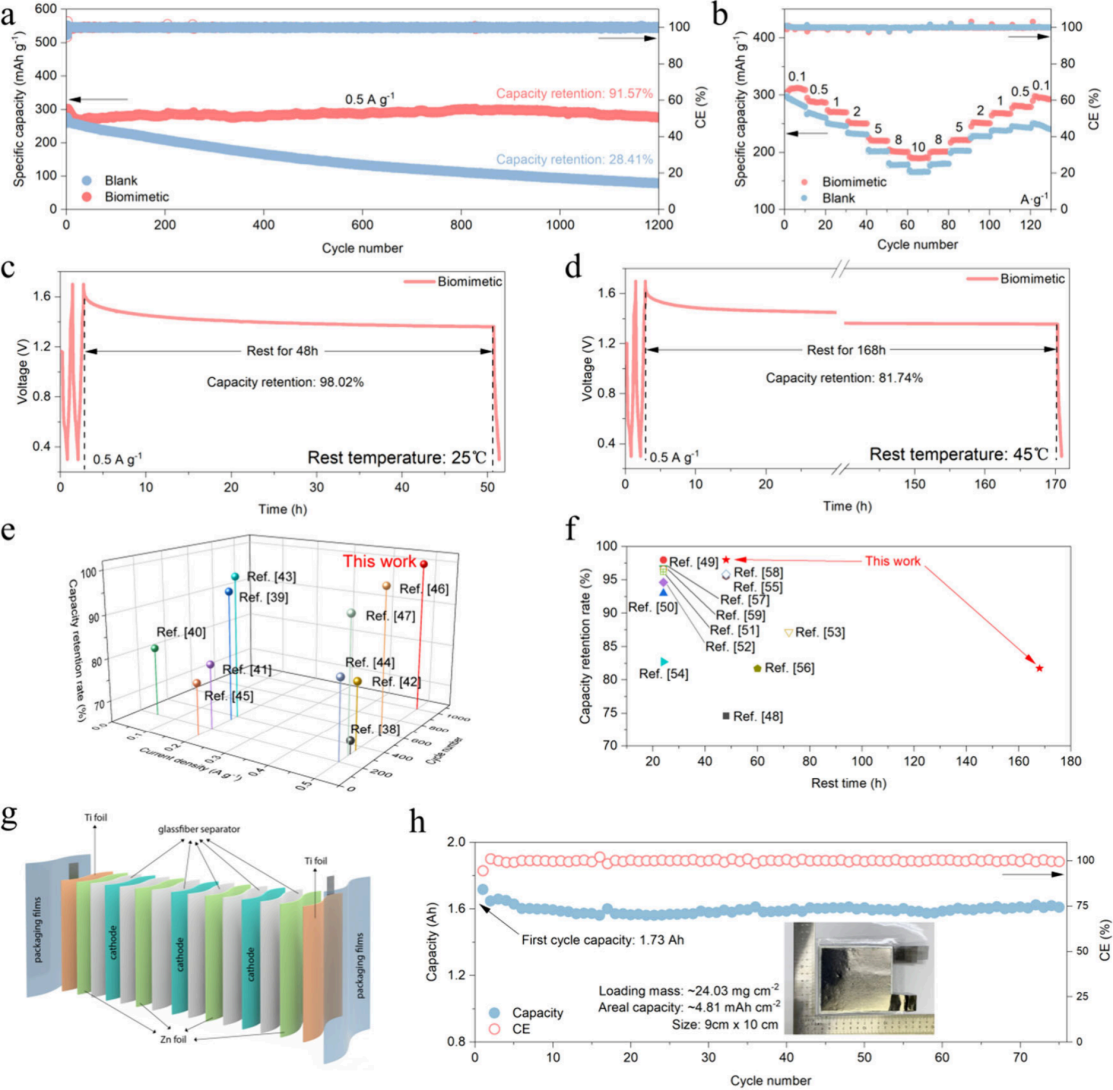
Performance of full batteries with biomimetic electrolytes. (a) Cycle performance of Zn||Zn_0.25_V_2_O_5_·*n*H_2_O with blank and biomimetic electrolytes at 0.5A g^–1^. (b) Rate performance of Zn||V_2_O_5_·H_2_O with blank and biomimetic electrolytes. Self-discharge measurement of a Zn||V_2_O_5_·H_2_O full cell with a biomimetic electrolyte: (c) rest for 48 h at 25 °C; (d) rest for 168 h at 45 °C. (e) Comparison of the full cell cycle performance under a low current density with vanadium-oxide-based cathode materials with previous studies.^34−43^ (f) Comparison of the rest performance with previous reports.^44−55^ (g) Schematic of the pouch cell. (h) Cycle performance of a 1.73 Ah Zn||Zn_0.25_V_2_O_5_·*n*H_2_O pouch cell with a biomimetic electrolyte (the inset is the image of the pouch cell).

To further study the benefits of biomimetic electrolytes for ZIBs, a Zn||Zn_0.25_V_2_O_5_·*n*H_2_O battery was assembled for testing. As shown in Figure 5a, the Zn||Zn_0.25_V_2_O_5_·*n*H_2_O battery with a biomimetic electrolyte shows a high capacity retention rate of 91.57% after 1200 cycles at 0.5 A g^–1^ (initial specific capacity, 301.5 mAh g^–1^; end specific capacity, 276.1 mAh g^–1^), while the battery with a blank electrolyte delivers a low capacity retention of only 28.41% (initial specific capacity, 275 mAh g^–1^; end specific capacity, 76.5 mAh g^–1^). Voltage profiles for the Zn||Zn_0.25_V_2_O_5_·*n*H_2_O battery can be seen in Figure S17. The corresponding cyclic voltammetry (CV) curves are also shown in Figure S18. Apparently, a more stable discharge peak appeared in the battery with a biomimetic electrolyte, proving the high stability of Zn_0.25_V_2_O_5_·*n*H_2_O. Because vanadium-based oxides are considered to be a cathode material that can operate stably for a long time in ZIBs, we believe that the difference in capacity retention between the two is mainly due to the reversibility of the zinc anode.^31,32^ During the cycling process in the blank electrolyte, the zinc anode is continuously consumed, and a large amount of byproducts accumulate, resulting in the tremendous consumption of zinc and rapid passivation of the zinc anode surface, making it difficult to continue to participate in the primary reaction of the battery and resulting in rapid capacity decay. Compared with that of the blank electrolyte, Zn||Zn_0.25_V_2_O_5_·*n*H_2_O full cells show a good rate performance with a biomimetic electrolyte, with better stability from 0.1 to 10 A g^–1^ (Figure 5b). In addition, the blank electrolyte was unable to return to its initial specific capacity after the rate test cycle, which was not observed in the biomimetic electrolyte.

The storage life of the battery is also a key factor in determining whether it can be a practical application. In ZIBs, the stability of the zinc metal anode is directly related to the storage life.^33^ Here, a biomimetic electrolyte was employed to assemble Zn||Zn_0.25_V_2_O_5_·*n*H_2_O full cells and storage life tests. The results show that the Zn||Zn_0.25_V_2_O_5_·*n*H_2_O full cell showed 98.02% capacity retention after resting for 48 h at 25 °C (Figure 5c) and 81.74% capacity retention after resting for 168 h at 40 °C (Figure 5d), which was significantly improved compared with the blank electrolyte, with only 52.62% capacity retention after storage for 48 h at 25 °C (Figure S19). Such an excellent performance can be ascribed to the superior stability of the zinc metal anode in the biomimetic electrolyte, which originates from the vigorous restriction of water activity by the biomimetic electrolyte and the stable zinc anode surface protective bilayer, thereby suppressing the spontaneous side reaction and avoiding self-discharge of the ZIBs during the rest. By a comparison of the performance of full cells with that reported in previous literature, the biomimetic electrolyte that we designed has great advantages in both cycling performance at low current density (Figure 5e)^34−43^ and rest life (Figure 5f).^44−55^

In order to further study the broad effect of a FBS anion-induced biomimetic electrolyte, we also added 3 M LiFBS to 2 M ZnSO_4_ + 0.2 M MnSO_4_ electrolyte to construct another kind of biomimetic electrolyte. The assembled Zn||MnO_2_ full cell using this electrolyte also exhibited excellent long-cycle performance of 1000 times with up to 100% capacity retention at 1 A g^–1^ (205.7 mAh g^–1^ before cycling and 207.8 mAh g^–1^ after cycling; Figures S20 and S21). Figure S22 shows the CV profiles of the Zn||MnO_2_ batteries. The good cyclability of Zn||MnO_2_ full cells proves that the FBS anion-induced biomimetic electrolyte has broad applicability.

For better practicality demonstration, a 4-fold 9 × 10 cm^2^ Zn||Zn_0.25_V_2_O_5_·*n*H_2_O pouch cell was assembled for testing. The schematic and image of the pouch cell are shown in Figure 5g,h. The assembled pouch cell shows a high capacity of 1.73Ah in the first cycle (loading mass, 24.03 mg cm^–2^; areal capacity, 4.81 mAh cm^–2^). After 75 cycles, the battery delivered a capacity of 1.61 Ah, showing good reversibility and high capacity retention (93%; Figure S23). The excellent performance of the pouch cell proves that the FBS anion-based biomimetic electrolyte also has good reversibility under practical conditions, proving that the biomimetic electrolyte is an effective way to improve the stability of the zinc anode and extend the lifespan of ZIBs.

## 3 Conclusion

In summary, inspired by the lipid bilayer from biology, we constructed a biomimetic electrolyte using LiFBS. The hydrophilic sulfonate groups on the outer and inner layer surfaces of the FBS ionic cluster particles anchor active H_2_O in the electrolyte, limiting their activity. Meanwhile, FBS anions lay flat on the surface of the zinc anode to form a lipid-bilayer-like protective structure and decompose during the electrochemical process to create a polyanion–ZnS_*x*_–ZnF_*x*_ organic–inorganic hybrid SEI, further stabilizing the zinc anode and protecting it from parasite reactions. Thanks to these effects, the performance of ZIBs is greatly improved. The Zn||Cu asymmetric battery has been cycled for more than 880 h at 5 mA cm^–2^ and 5 mAh cm^–2^ with an average CE of 99.91%. The Zn||Zn symmetrical battery was cycled for 2460 h at 5 mA cm^–2^ and 5 mAh cm^–2^, with a cumulative capacity of 6 Ah cm^–2^. Both Zn||Zn_0.25_V_2_O_5_·*n*H_2_O and Zn||MnO_2_ full cells employing biomimetic electrolytes cycled 1000 times at 0.5 A g^–1^ and 1 A g^–1^, respectively, and the capacity retention was close to 100%. Besides, Zn||Zn_0.25_V_2_O_5_·*n*H_2_O full cells demonstrate an excellent storage life of 81.74% capacity retention after placement for 7 days. Moreover, the Zn||Zn_0.25_V_2_O_5_·*n*H_2_O pouch cell delivered a high capacity of 1.73 Ah and a high areal capacity of 4.81 mAh cm^–2^, demonstrating a certain practicality. Our research indicates that biomimetic electrolytes are an efficient measure to improve the reversibility of zinc anodes and boost the performance of ZIBs for further practical applications.

## 4 Methods

### Electrolyte Preparation

All of the salts and solvents used in the electrolytes were bought from Aladdin and used without further purification. The biomimetic electrolyte was prepared by dissolving 3 M Zn(Otf)_2_ and 3 M LiFBS into the deionized (DI) water. The blank electrolyte is a pure 3 M Zn(Otf)_2_ electrolyte. 1 M LiFBS and 2 M LiFBS electrolytes are fabricated by dissolving 1 M LiFBS and 2 M LiFBS to 3 M Zn(Otf)_2_, respectively.

### Preparation of Cathode Materials

Zn_0.25_V_2_O_5_·*n*H_2_O was synthesized according to a previous study.^56^ Specifically, 3.492 g of V_2_O_5_ (Aladdin) was dispersed in 480 mL of a 15:1 water/acetone mixture solution. A total of 2.3 g of anhydrous zinc acetate (Aladdin) was added to the solution and stirred for 2 h. The mixed solution was then transferred to several sealed Teflon vessels and heated for 72 h. The obtained product was centrifuged and washed three times with water and isopropyl alcohol, respectively, and finally dried under vacuum for 24 h to obtain a green solid.

MnO_2_ was bought from SAIBO Electrochemical Reagents and Materials.

### Electrode Fabrications

#### Coin Cells

Stainless steel cases of coin cells (CR2032) were bought from KELUDE. A stamping machine prepared electrodes for the coin cells. To minimize the impact of the electrolyte on the corrosion of the stainless steel battery cases, the Cu foil current collector in the Zn||Cu asymmetric cells was stamped into 18-mm-diameter flakes. Zinc metal anodes were stamped into 14-mm-diameter flakes.

#### Pouch Cells.

To prepare the Zn_0.25_V_2_O_5_·*n*H_2_O electrode for the pouch cell, Zn_0.25_V_2_O_5_·*n*H_2_O powders were mixed with Super P and a PTFE–H_2_O solution with a weight ratio of about 7:2:1. The mixed slurry was evenly coated on both sides of the 9 × 10 cm^2^ 40-mesh Ti mesh. The obtained electrode was dried under a vacuum for 24 h. Similarly, the zinc foil wass cut into pieces of 9 × 10 cm^2^ size to be the zinc metal anode.

### Characterization

The FTIR spectra were collected from a PerkinElmer Spectrum II FT-IR spectrometer. The Raman spectra were collected from a WITec RAMAN Alpha 300R spectrometer, and the laser source was 532 nm. The SEM images were collected from an Environmental Scanning Electron Microscope (FEI/Philips XL30 Esem-FEG). The ex situ SEM samples were obtained by disassembling the cell, extracting the electrode, washing three times with a DI water/ethanol 50% mixture, and drying in a vacuum (∼25 °C) for 24 h. DLS measurements were performed on a DLS instrument (Zetasizer Nano ZS, Malvern Instruments Ltd.) at 25 °C with a scattering angle of 90° and a laser wavelength of 632.8 nm. Typically, the gas evolution volume was measured by a self-designed equipment of our group that was previously reported.^22^

## Supporting Information Available

Computational details, zinc consumption rate calculation method, and Figures S1–S23 (PDF)
