# Supplementary material for: Constructing Lipid-Like Biomimetic Structure via Electrolyte Designation for Stable Zinc-Ion Batteries
Source: ACS Nano. 2025 Apr 7;19(14):14085–96. doi: 10.1021/acsnano.4c18796 (PMC12005047; doi:10.1021/acsnano.4c18796)
Supplement: Supplementary file 1 — nn4c18796_si_001.pdf [file nn4c18796_si_001.pdf]

Supplementary information

Constructing lipid-like biomimetic structure via electrolyte designation for stable zinc ion batteries

Zhuoxi Wu[1], Shuo Yang[1], Zhiquan Wei[1], Yiqiao Wang[1], Xinru Yang[1], Jiaxiong Zhu[1], Hu Hong[1], Pei Li[1], Xue-Feng Yu[2], Chao Peng[2*], Chunyi Zhi[1,3,4,5*]

1 Department of Materials Science and Engineering, City University of Hong Kong, Kowloon, 999077, Hong Kong SAR, China

2 Materials Interfaces Center, Shenzhen Institute of Advanced Technology, Chinese Academy of Sciences, Shenzhen 518055, Guangdong, China

3 Hong Kong Institute for Advanced Study, City University of Hong Kong, Kowloon, 999077, Hong Kong SAR, China

4 Hong Kong Institute for Clean Energy, City University of Hong Kong, Kowloon, 999077, Hong Kong SAR, China

5 Centre for Advanced Nuclear Safety and Sustainable Development, City University of Hong Kong, Kowloon, 999077, Hong Kong SAR, China

*E-mail: chao.peng@siat.ac.cn, cy.zhi@cityu.edu.hk

Computational details

The molecular dynamics (MD) simulations were performed to model the electrolyte compositions containing $Zn(Otf)_2$, LiFBS and H_2O utilizing the LAMMPS software.[1] The force field parameters of Zn^{2+}, OTf^-, Li^+ and FBS^- are obtained from the literature.[2–4] H_2O molecules were described by the SPC/E model.[3] All the simulations were conducted with initial energy minimization for structure optimization and followed by a pre-equilibrated period within the NpT ensemble at 298.15 K with a timeline of 1 ns and finally carried out within the NVT ensemble at

298.15 K with a sampling time of 5 ns. The time step is 1.0 fs. The analyses of radial distribution functions (RDF) and coordination number (CN) were captured from the last 2 ns. The $Zn(Otf)_2$, LiFBS and H_2O molecules were randomly introduced to construct the electrolyte mixture using Packmol software.[5] The Moltemplate software was adopted to construct the inputs for LAMMPS.[6] The density functional theory (DFT) calculations were executed utilizing the Vienna Ab initio Simulation Package (VASP).[7,8] The Generalized Gradient Approximation (GGA) was applied, with the Perdew–Burke–Ernzerhof (PBE) function chosen for treating the exchange-correlation interactions.[9] The projector-augmented wave (PAW) method described the core electrons, while valence electronic states were expanded using plane-wave basis sets.[10] Dispersion interactions were handled using the DFT-D3 method with Becke–Johnson (BJ) damping.[11,12] The cutoff energy was defined as 450 eV, and the force convergence criterion was set to 0.05 eV/Å. The adsorption energy (Eads) was calculated with the equation as follows:

$$E_{ads}= E(Zn|adsorbate) – E(Zn) – E(species)$$

where E(Zn|adsorbate), E(Zn) and E(species) represent the total energies of the adsorption structure, the clean Zn(001) slab and adsorbed species, respectively.

Zinc consumption rate calculation

In short, since the electrochemical testing system cannot distinguish the electrons provided by the reversible and irreversible side reactions, they will all be included in calculating the CE. During the cycling process, zinc can be consumed reversibly or irreversibly (such as the formation of irreversible byproducts on the electrode surface or irreversible dissolution). Therefore, calculating the zinc consumption rate can more intuitively reflect the reversibility of the zinc negative electrode. When zinc is consumed in large quantities and cannot provide sufficient

capacity, a corresponding inflection point will occur on the capacity curve. The zinc consumption rate can be calculated based on the position of the inflection point:

$$\eta = \frac{\left(\frac{D}{1.7} - C_i\right)}{N} / C_0 \text{..................equation 1}$$

Where D is the thickness of zinc foil, C_i is the areal capacity at the inflection point, N is the cycle number, and C_0 is the areal capacity set for cycling.

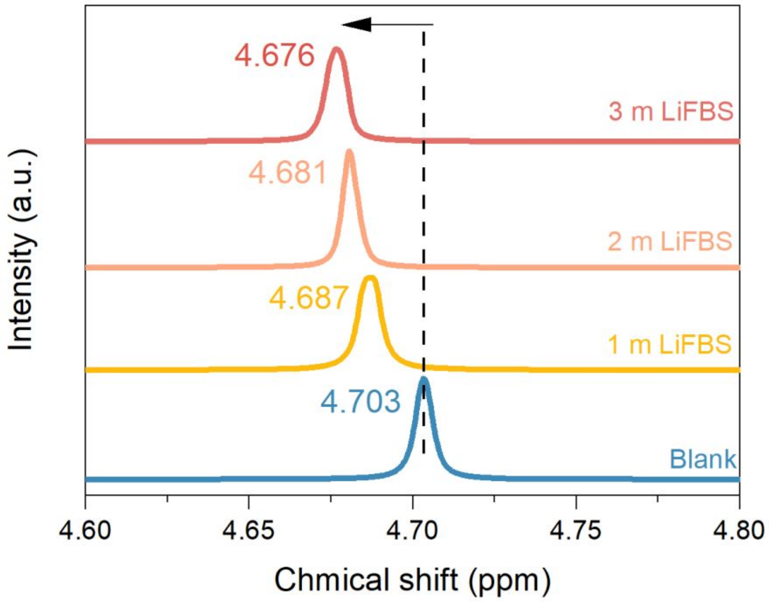

Figure S1. NMR spectra of different electrolytes (from bottom to top is blank 3 m $Zn(Otf)_2$, 3 m $Zn(Otf)_2$ + 1 m LiFBS, 3 m $Zn(Otf)_2$ + 2 m LiFBS, 3 m $Zn(Otf)_2$ + 3 m LiFBS, respectively).

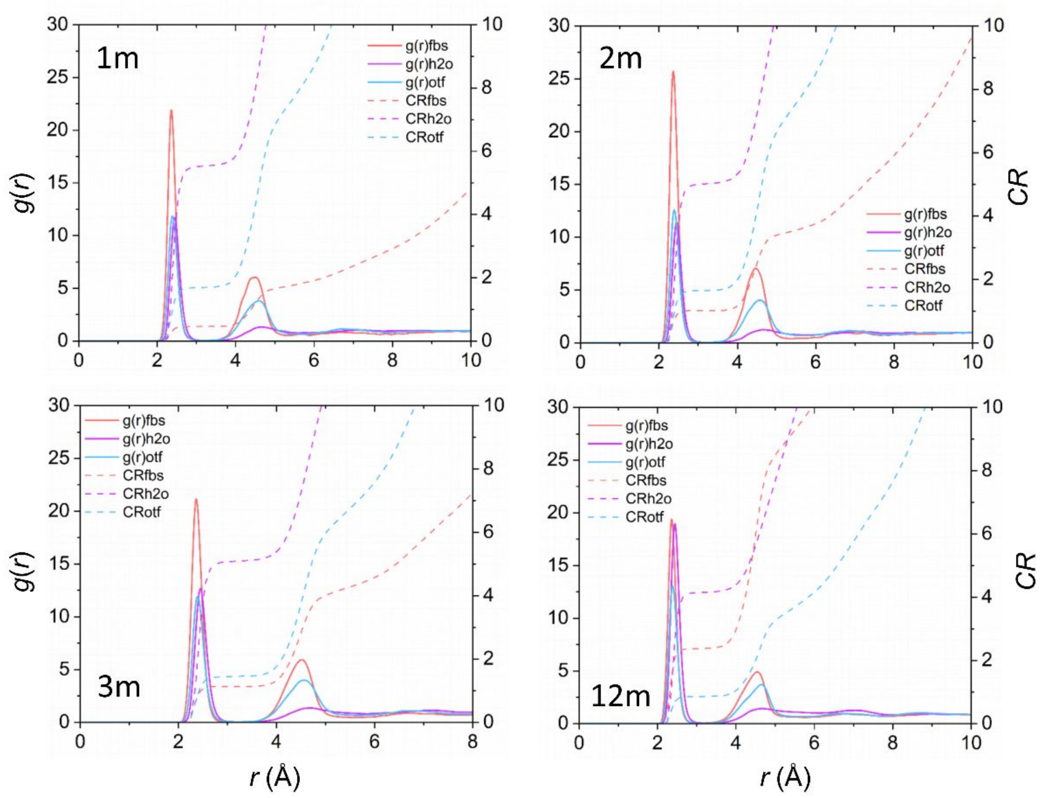

Figure S2. MD simulation of 3m Zn(Otf)$_2$ + xm LiFBS (x=1, 2, 3, 12). (a-d) The Zn-H_2O, Zn-Otf$^-$ and Zn-FBS$^-$ radial distribution function (g(r)) and coordination numbers (c(r)). Results indicate that as the concentration of LiFBS increases, FBS$^-$ anions would substitute the H_2O and Otf$^-$ anions in the Zn^{2+} solvation sheath.

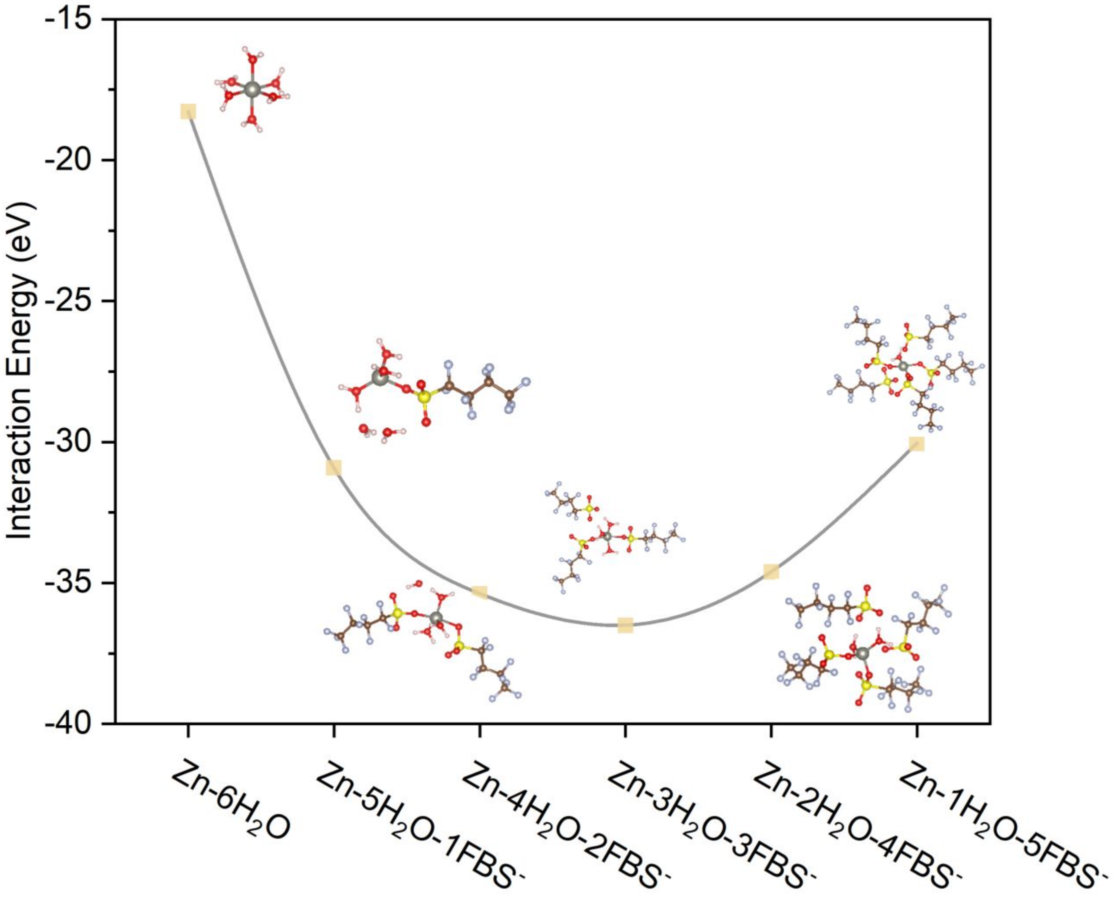

Figure S3. DFT results of the primary solvation shell and corresponding interaction energy of $Zn^{2+}(H_2O)n(FBS^-)_{6-n}$ (n=6, 5, 4, 3, 2, 1, 0).

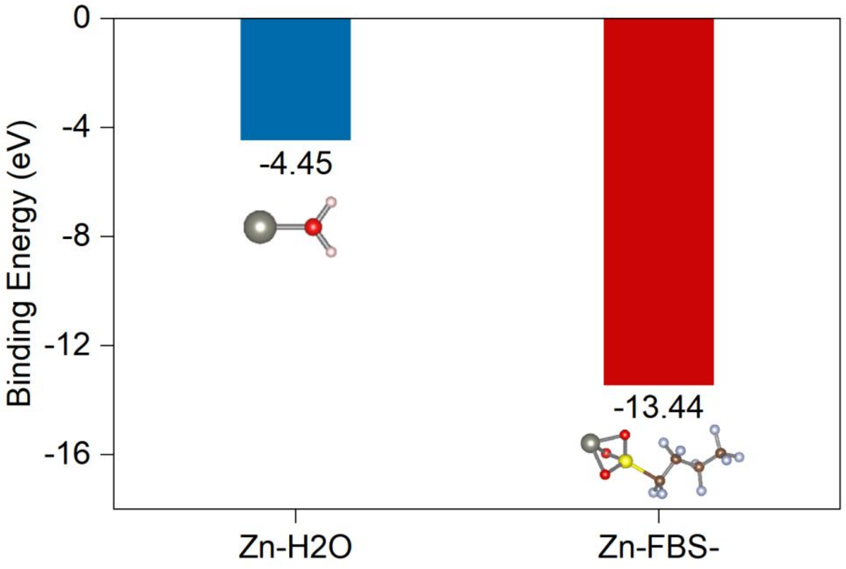

Figure S4. Binding energy of Zn^{2+} - H_2O and Zn^{2+} - FBS^{-}.

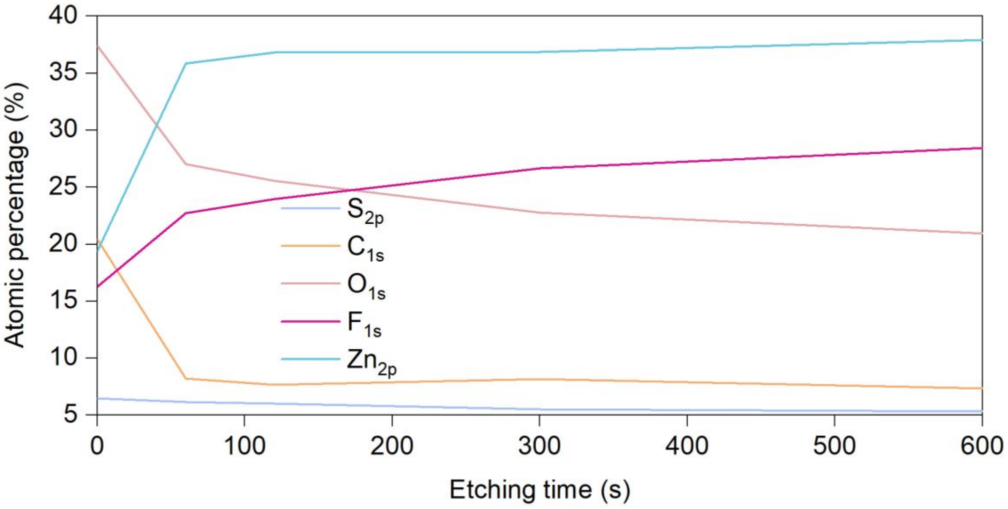

Figure S5. Atomic percetage at different etching time.

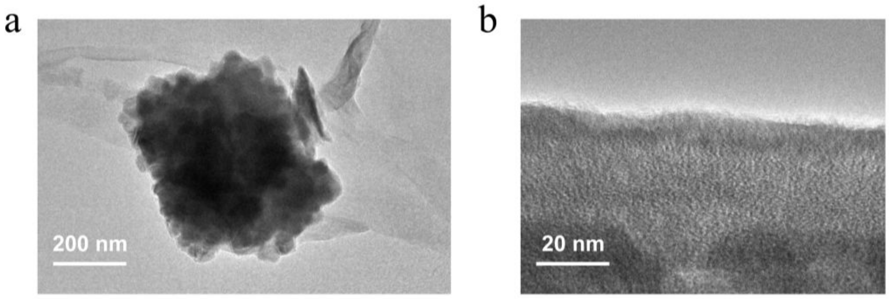

Figure S6. TEM images of zinc cycled in blank electrolyte (a) zinc particles under TEM observation, and (b)the enlarged edge of the particle.

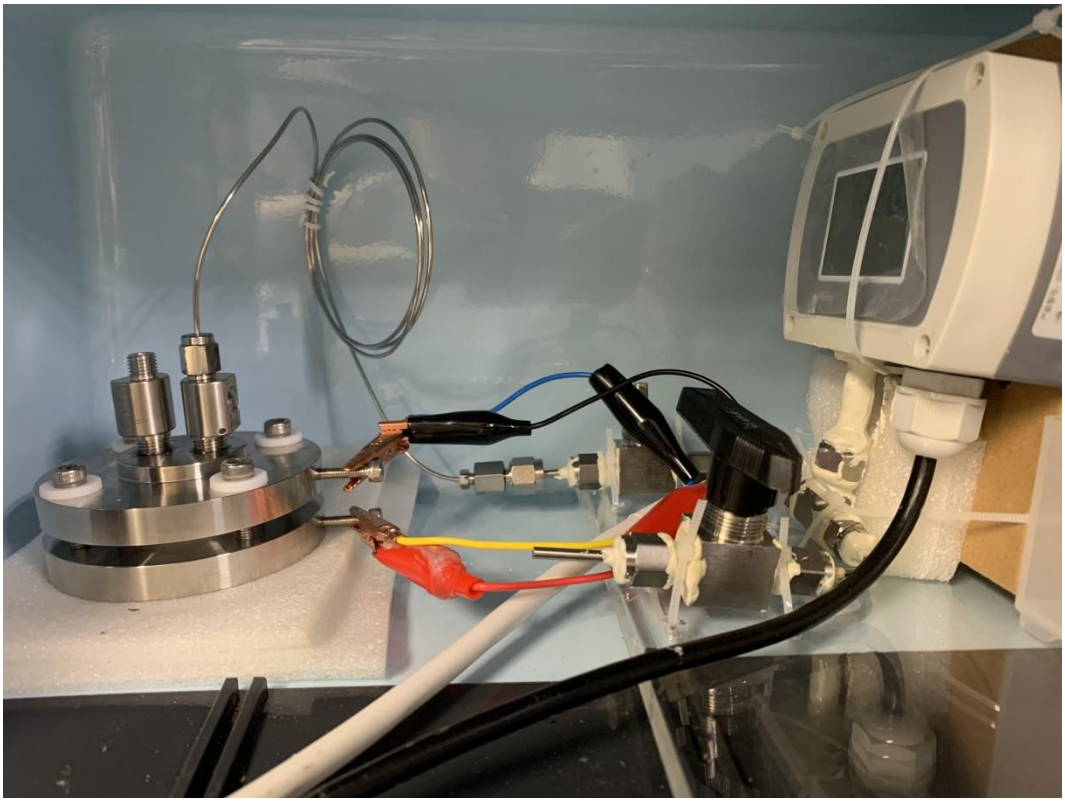

Figure S7. Image of gas collecting equipment. From left to right are the stainless steel current collector, gas conduit, and pressure sensing device. For detailed structure, please refer to the work previously reported by our group.[3]

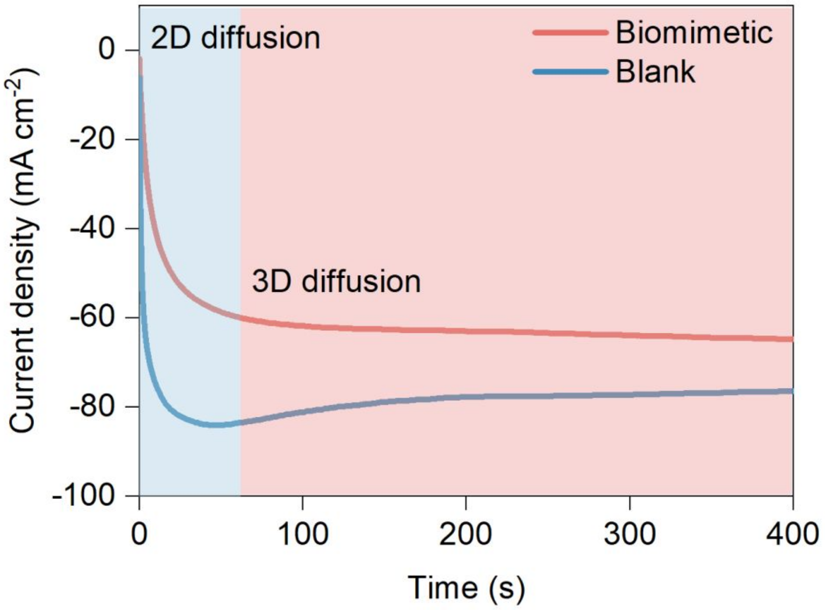

Figure S8. Chronoamperometry testing of Zn||Zn symmetric batteries with blank and

biomimetic electrolytes.

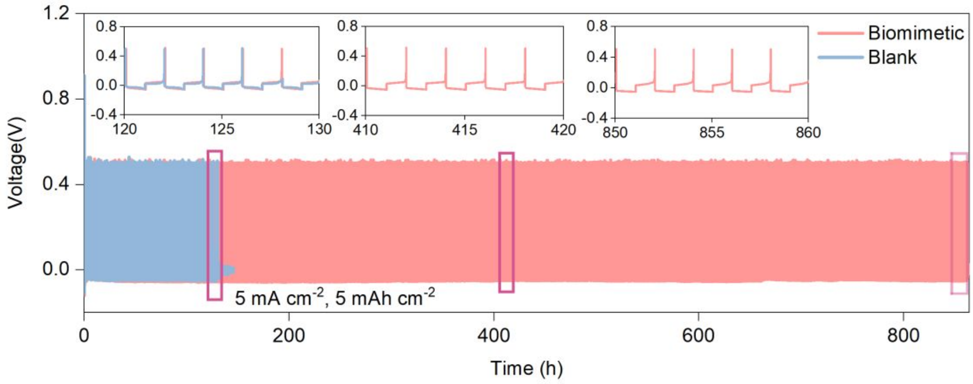

Figure S9. Time-Voltage curves of Zn||Cu asymmetric cells with blank electrolyte and biomimetic electrolyte at 5 mA cm^{-2}, 5 mAh cm^{-2}, the insets show the representative voltage profile.

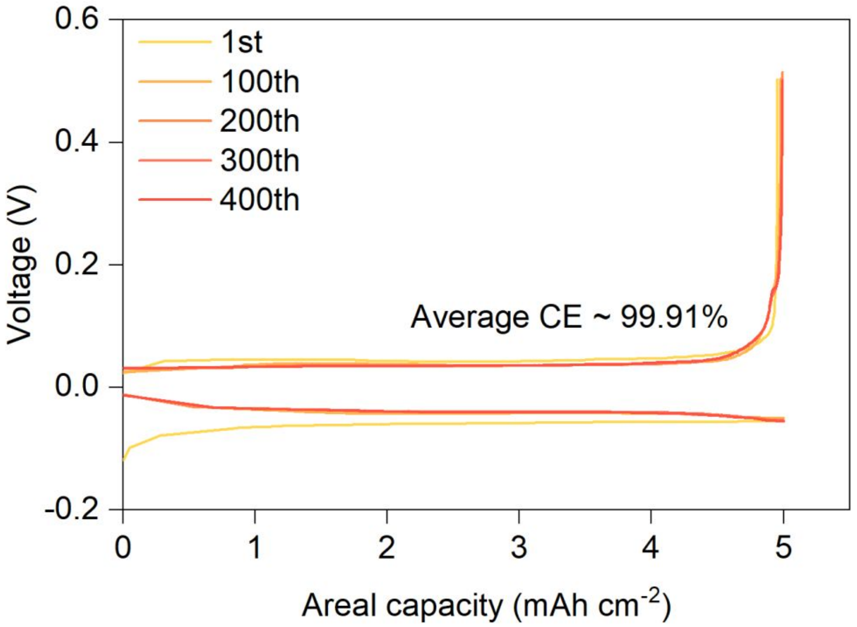

Figure S10. Voltage profiles of Zn||Cu half cell at 1st, 100th, 200th, 300th, 400th, respectively.

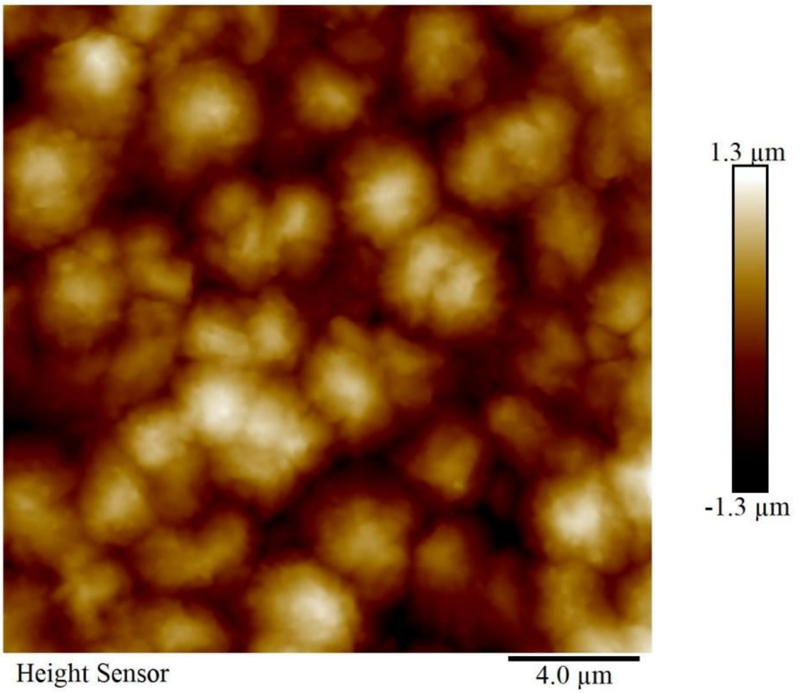

Figure S11. AFM contour map of zinc anode after cycling in blank electrolyte.

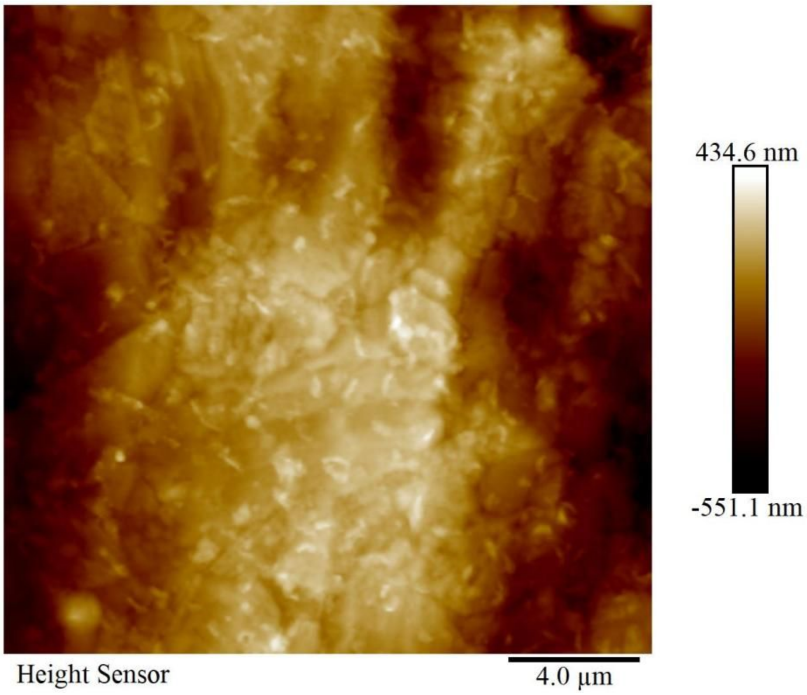

Figure S12. AFM contour map of zinc anode after cycling in biomimetic electrolyte.

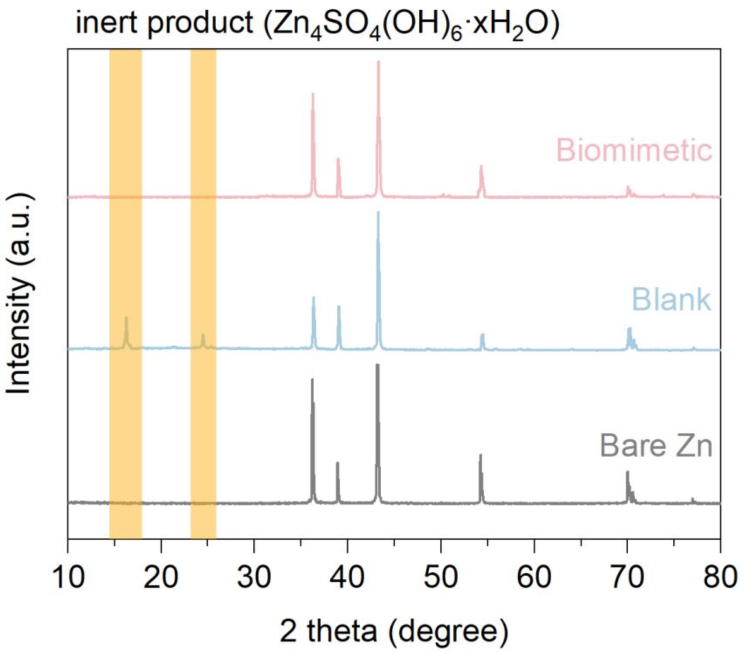

Figure S13. XRD result of zinc anode after cycle.

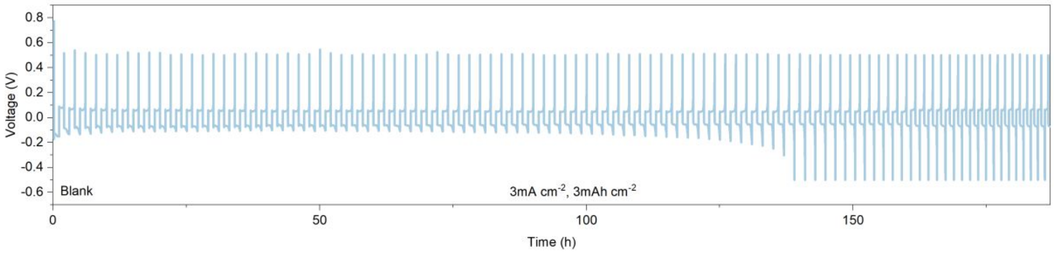

Figure S14. Time-voltage curves of Zn||Cu half cell with blank electrolyte at 3 mA cm^{-2}, 3 mAh cm^{-2}.

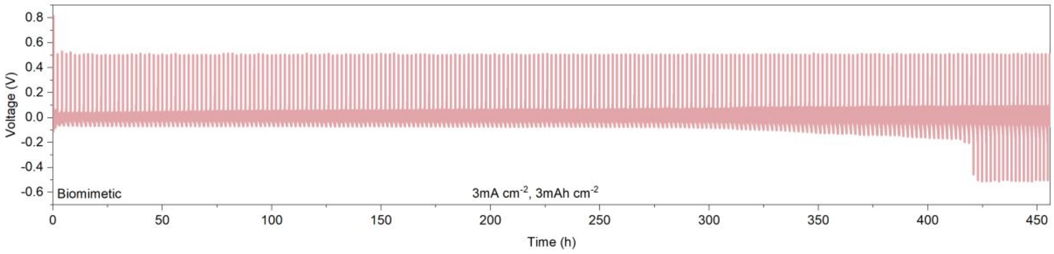

Figure S15. Time-voltage curves of Zn||Cu half cell with biomimetic electrolyte at 3 mA cm^{-2}, 3 mAh cm^{-2}.

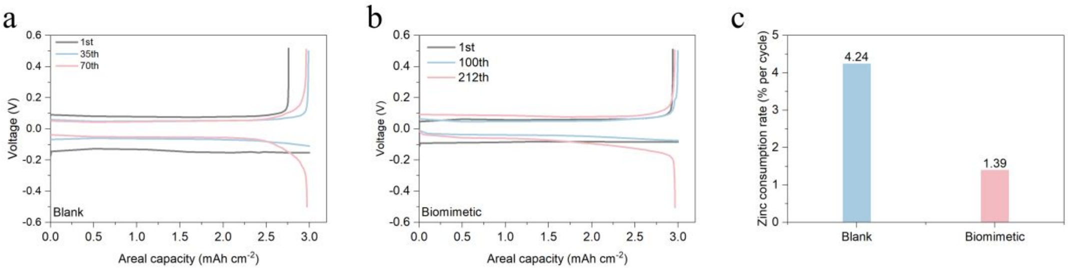

Figure S16. Voltage profiles at different cycle of (d) blank electrolyte and (e) biomimetic electrolyte, respectively. (f) Comparison of zinc consumption rate in average.

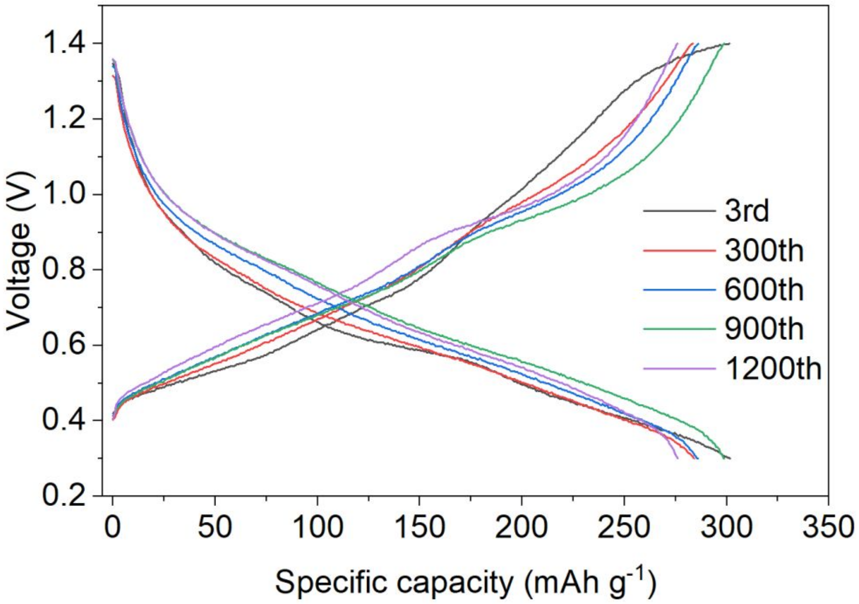

Figure S17. Voltage profiles of Zn||$Zn_{0.25}V_2O_5 \cdot nH_2O$ full cells at different cycles.

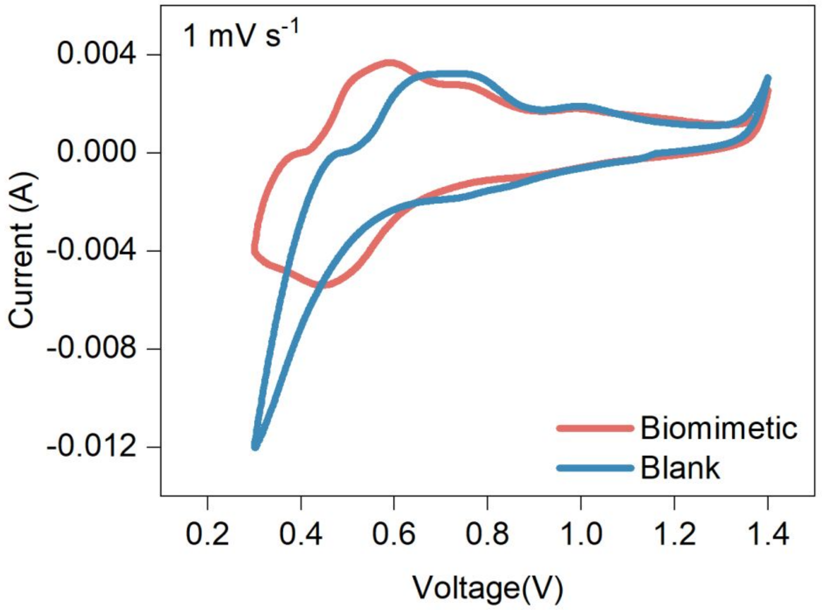

Figure S18. Cyclic voltammetry profile of Zn||$Zn_{0.25}V_2O_5$ · nH_2O with biomimetic electrolytes and blank electrolyte.

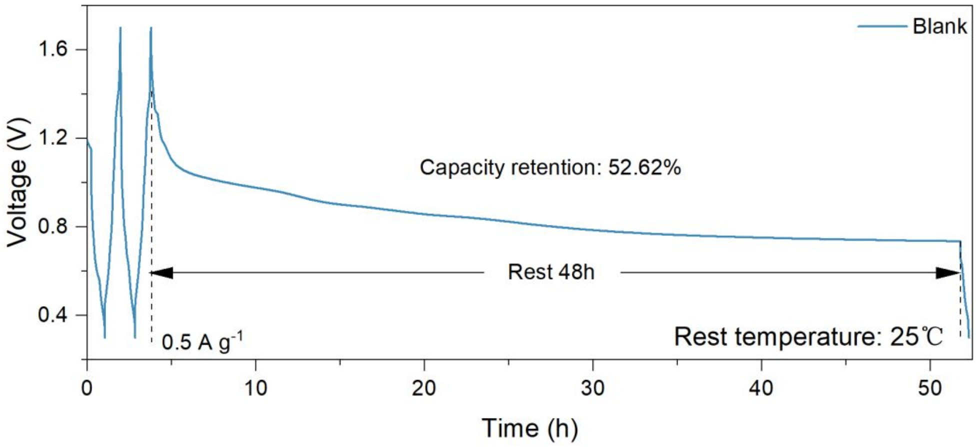

Figure S19. Storage life of Zn||$Zn_{0.25}V_2O_5$ · nH_2O full cell with blank electrolyte after 48h.

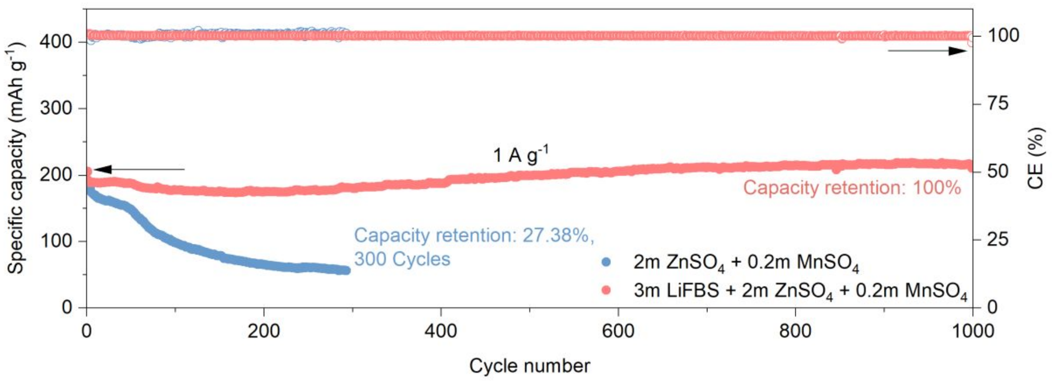

Figure S20. Cycle performance of Zn||MnO$_2$ full cell with blank ZnSO$_4$ electrolyte and 2m ZnSO$_4$ + 3m LiFBS electrolyte.

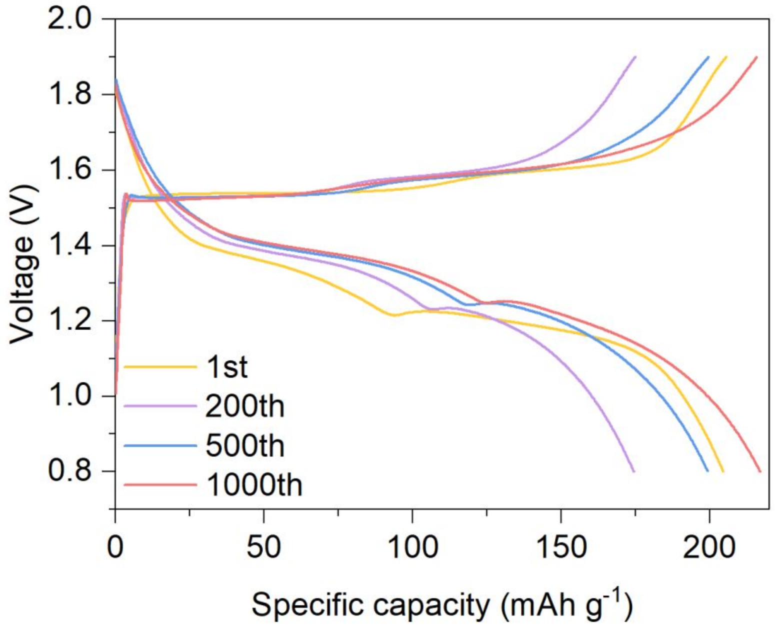

Figure S21. Voltage profile of Zn||MnO2 full cell at 1st, 200th, 500th, 1000th cycle with 2m ZnSO$_4$ + 3m LiFBS electrolyte.

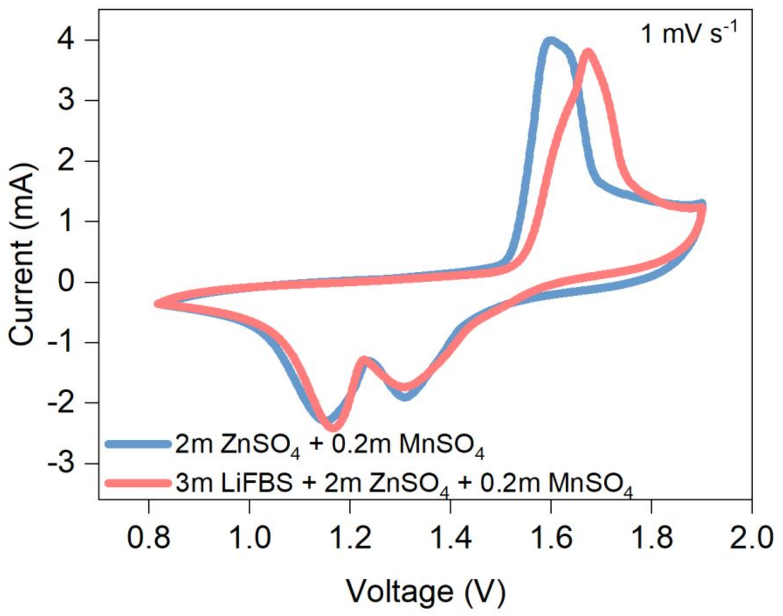

Figure S22. Cyclic voltammetry profile of Zn||MnO_2 with 3m LiFBS + 2m $ZnSO_4$ + 0.2m $MnSO_4$ and 2m $ZnSO_4$ + 0.2m $MnSO_4$ electrolyte.

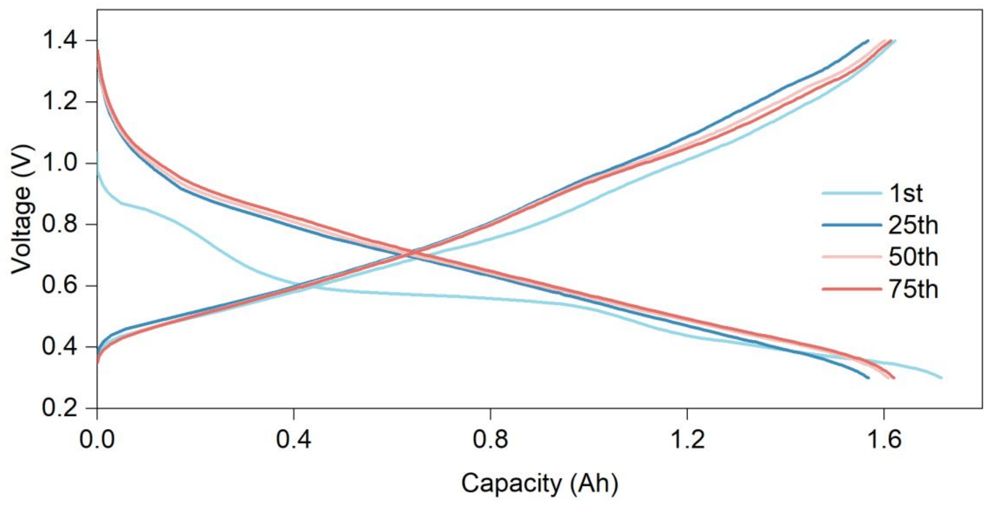

Figure S23. Voltage profile of Zn||$Zn_{0.25}V_2O_5 \cdot nH_2O$ pouch cell at 1st, 25th, 50th, 75th cycle.

Reference

(1) Thompson, A. P.; Aktulga, H. M.; Berger, R.; Bolintineanu, D. S.; Brown, W. M.; Crozier, P. S.; in 't Veld, P. J.; Kohlmeyer, A.; Moore, S. G.; Nguyen, T. D.; Shan, R.; Stevens, M. J.; Tranchida, J.; Trott, C.; Plimpton, S. J. LAMMPS - a Flexible Simulation Tool for Particle-Based Materials Modeling at the Atomic, Meso, and Continuum Scales. *Comput Phys Commun* **2022**, *271*. https://doi.org/10.1016/j.cpc.2021.108171.

(2) Dezfoli, A. A.; Mehrabian, M. A.; Hashemipour, H. Molecular Dynamics Simulation of Heavy Metal Ions in Aqueous Solution Using Lennard-Jones 12-6 Potential. *Chem Eng Commun* **2015**, *202* (12), 1685–1692. https://doi.org/10.1080/00986445.2014.970251.

(3) Wang, Y.; Wang, T.; Bu, S.; Zhu, J.; Wang, Y.; Zhang, R.; Hong, H.; Zhang, W.; Fan, J.; Zhi, C. Sulfolane-Containing Aqueous Electrolyte Solutions for Producing Efficient Ampere-Hour-Level Zinc Metal Battery Pouch Cells. *Nat Commun* **2023**, *14* (1). https://doi.org/10.1038/s41467-023-37524-7.

(4) Patra, S.; Thakur, P.; Soman, B.; Puthirath, A. B.; Ajayan, P. M.; Mogurampelly, S.; Karthik Chethan, V.; Narayanan, T. N. Mechanistic Insight into the Improved Li Ion Conductivity of Solid Polymer Electrolytes. *RSC Adv* **2019**, *9* (66), 38646–38657. https://doi.org/10.1039/c9ra08003a.

(5) Martinez, L.; Andrade, R.; Birgin, E. G.; Martínez, J. M. PACKMOL: A Package for Building Initial Configurations for Molecular Dynamics Simulations. *J Comput Chem* **2009**, *30* (13), 2157–2164. https://doi.org/10.1002/jcc.21224.

(6) Jewett, A. I.; Stelter, D.; Lambert, J.; Saladi, S. M.; Roscioni, O. M.; Ricci, M.; Autin, L.; Maritan, M.; Bashusqeh, S. M.; Keyes, T.; Dame, R. T.; Shea, J. E.; Jensen, G. J.; Goodsell, D. S. Moltemplate: A Tool for Coarse-Grained Modeling of Complex Biological Matter and Soft Condensed Matter Physics. *J Mol Biol* **2021**, *433* (11). https://doi.org/10.1016/j.jmb.2021.166841.

(7) Kresse, G.; Furthmiiller B ', J. *Efficiency of Ab-Initio Total Energy Calculations for Metals and Semiconductors Using a Plane-Wave Basis Set*; 1996; Vol. 6.

(8) Kresse, G.; Furthmü, J. *Efficient Iterative Schemes for Ab Initio Total-Energy Calculations Using a Plane-Wave Basis Set*; 1996.

(9) Perdew, J. P.; Burke, K.; Ernzerhof, M. *Generalized Gradient Approximation Made Simple*; 1996.

(10) Kresse, G.; Joubert, D. *From Ultrasoft Pseudopotentials to the Projector Augmented-Wave Method*.

(11) Grimme, S.; Ehrlich, S.; Goerigk, L. Effect of the Damping Function in Dispersion Corrected Density Functional Theory. *J Comput Chem* **2011**, *32* (7), 1456–1465. https://doi.org/10.1002/jcc.21759.

(12) Grimme, S.; Antony, J.; Ehrlich, S.; Krieg, H. A Consistent and Accurate Ab Initio Parametrization of Density Functional Dispersion Correction (DFT-D) for the 94 Elements H-Pu. *Journal of Chemical Physics* **2010**, *132* (15). https://doi.org/10.1063/1.3382344.
